# Morphological relationships between external auditory canal and vital structures of tympanic cavity

**DOI:** 10.1186/s13005-022-00341-2

**Published:** 2022-11-18

**Authors:** Shinya Ohira, Manabu Komori, Mitsuto Nakamura, Kentaro Matsuura, Hiroshi Osafune, Riko Kajiwara, Kota Wada

**Affiliations:** 1 Department of Otolaryngology, Shonan Kamakura General Hospital, 1370-1, Okamoto, Kamakura-shi, Kanagawa 247–8533 Japan; 2 Department of Otolaryngology, Toho University Omori Medical Center, Omori, Tokyo Japan; 3 Department of Otolaryngology, St. Marianna University, Kawasaki, Kanagawa Japan

**Keywords:** External auditory canal, Tympanic sinus, Facial nerve, Three-dimensional computed tomography reconstruction

## Abstract

**Purpose:**

We aimed to evaluate the morphology of the external auditory canal (EAC) using a three-dimensional (3D) reconstruction of computed tomography (CT) scans of the temporal bone to corroborate and predict important anatomical structures involved in middle ear surgery based on the EAC morphology.

**Methods:**

Temporal bone CT from 62 patients (120 ears) was used to perform 3D reconstruction (maximum intensity projection), of which 32 patients (60 ears) had chronic otitis media and 30 patients (60 ears) had normal temporal bones. The anatomical morphology of the EAC, tympanic sinus, vertical portion of the facial nerve, and jugular bulb were measured, and the anatomical relationship between the EAC morphology and important structures of the middle ear was analyzed.

**Results:**

In ears with chronic otitis media, the overhang of the inferior wall of the EAC was significantly more than that in normal ears, and the antero-posterior length of the bony tympanic ring was short. Furthermore, the tympanic sinus was shallow, and vertical portion of the facial nerve tended to run outward. The EAC morphology correlated with the tympanic sinus depth and outward orientation of the vertical portion of the facial nerve.

**Conclusion:**

A severe overhang of the inferior wall of the EAC and short antero-posterior length of the bony tympanic ring indicates a higher possibility of a shallow tympanic sinus and an outward orientation of the vertical portion of the facial nerve. These findings aid in predicting the difficulty of tympanic sinus operation and reducing facial nerve damage risk during EAC excision.

## Introduction

The morphology of the external auditory canal (EAC) is the first structure to be visually confirmed in the diagnosis of ear diseases; moreover, middle ear diseases are often surgically treated via the EAC. There are individual variations in the morphology of the EAC, and these differences limit the ease of inspection and the range of possible operations [1]. The introduction of endoscopes in recent years has made it possible to obtain a clear field of view even in cases of EAC curvature; however, sometimes it may be necessary to shave the EAC bone in order to perform sufficient operations. In such instances, it is necessary to exercise caution to prevent damage to important structures. If the morphology of the tympanic sinus, which is a difficult-to-operate area in the middle ear, and the morphology of the vertical portion of the facial nerve and the jugular bulb, which are prone to collateral damage, can be inferred from the morphology of the EAC, it would be useful for the prediction of the morphology of the middle ear during the medical examination. However, there have been no reports examining these relationships. In this study, we investigated these anatomical structural relationships to predict the morphology of the vital structures in the middle ear based on the EAC morphology.

## Methods

This study examined 32 patients (60 ears) diagnosed with chronic perforated otitis media and 30 patients (60 ears) that underwent temporal bone computed tomography (CT) owing to symptoms such as sensorineural hearing loss, facial paralysis, and dizziness, at the Shonan Kamakura General Hospital from April 2019 to March 2022.

Children under the age of 18 years, patients with trauma, congenital malformations, auditory canal cholesteatoma, and acquired cholesteatoma, and non-Asian cases were excluded because in these cases the EAC morphology may have been affected for other reasons. All patients provided informed consent, and the study protocol was reviewed and approved by the relevant Institutional Review Board (Approval No. TGE01880-024). Anatomical structures were evaluated using the temporal bone CT. Multi-slice temporal CT data were obtained from 80-row and 320-row detector CT (Canon Medical Systems, Tochigi, Japan). The slice thickness was 0.5 mm, and the scanning parameters were 160 mA and 120 kV.

### Morphology of the tympanic sinus

**Fig. 1:**
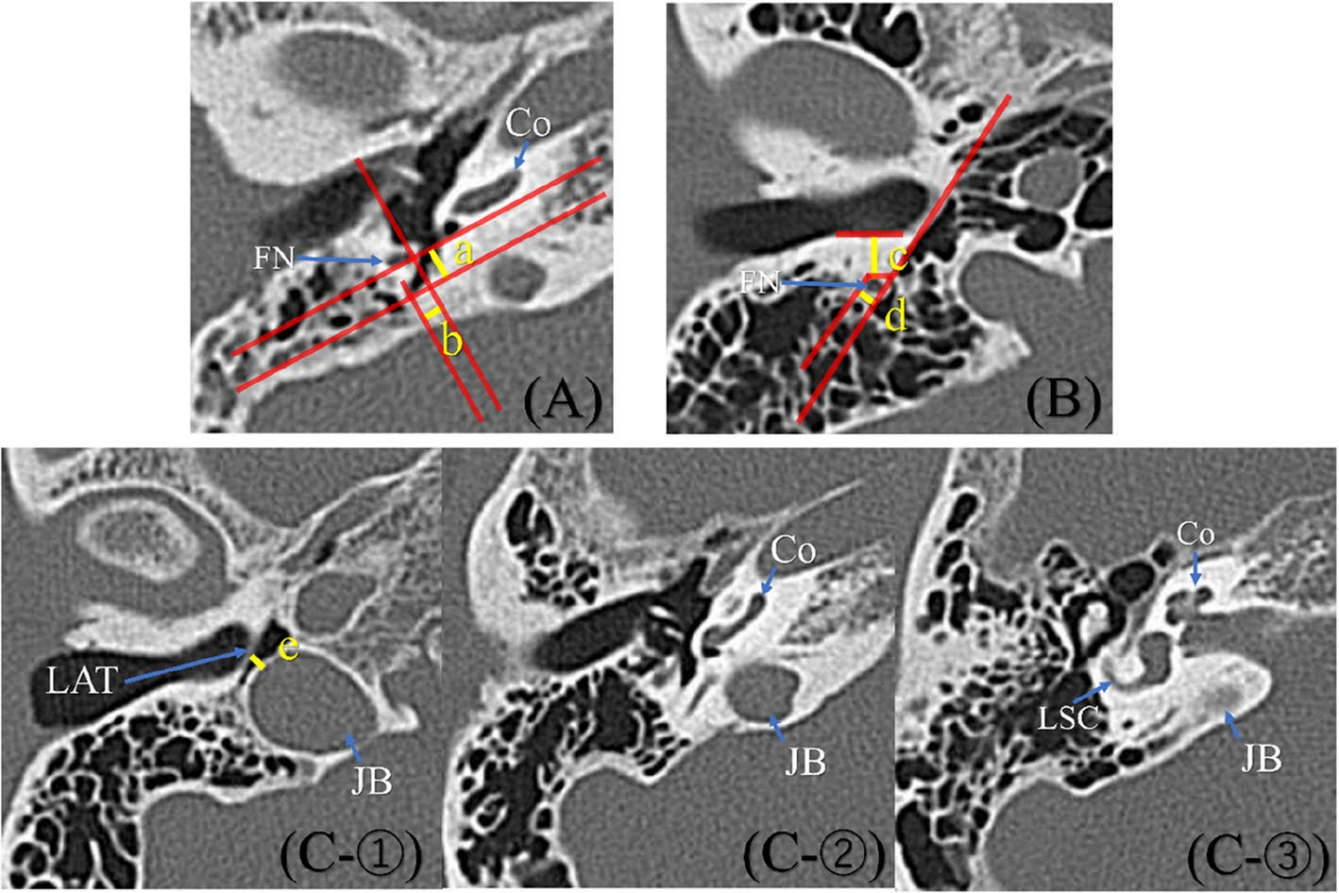
Morphological evaluation of the tympanic sinus, vertical portion of the facial nerve, and jugular bulb. A shows the posterior (a) and outer (b) distance of the tympanic sinus compared with straight lines drawn horizontal to the basal turn of the cochlea and touching the posterior portion of the facial nerve. Another straight line drawn perpendicular to the first line touches the interior of the facial nerve using slice where growth of the tympanic sinus cavity can be typically confirmed. B shows the posterior distance from the line of the posterior wall of the external auditory canal to the vertical portion of the facial nerve (c), and the lateral distance from the tympanic membrane line to the vertical portion of the facial nerve (d) using slices at a height midway between the tympanic umbo and the lower edge of the bony tympanic ring. C shows the shortest distance (e) from the tympanic membrane to the jugular bulb at the height of the lower edge of the tympanic ring (C-①). The height of the jugular bulb (f) was measured from the number of slices from the bottom of the jugular bulb(C-①) to the apex of the jugular bulb(C-③), multiplied by the slice width

The growth of the tympanic sinus was classified as follows, by drawing a straight line that was horizontal to the basal turn of the cochlea and touched the posterior portion of the facial nerve; another straight line was drawn perpendicular to the first line and touched the interior of the facial nerve (Fig. 1 A).

Class 1: Growth toward a place interior to the interior line of the facial nerve and shallower than the posterior line.

Class 2: Growth toward a place interior to the interior line of the facial nerve and deeper than the posterior line.

Class 3: Growth toward a place more lateral than the interior line of the facial nerve.

Regarding the depth of the tympanic sinus, the posterior distance from the posterior line to the deepest part (a) and the lateral distance from the inner line to the deepest part (b) were measured. Class 1 was judged to be 0 mm for both distances.

### Morphology of the vertical portion of the facial nerve

Using the axial slices (Fig. 1B) at a height between the tympanic umbo and the inferior edge of the bony tympanic ring, the posterior distance from the line of the posterior wall of the EAC (c) and the lateral distance from the line of the tympanic membrane to the vertical portion of the facial nerve (d) was measured.

### Morphology of the jugular bulb

Upon confirming the inferior edge of the bony tympanic membrane in the axial slices, cases wherein the apex of the jugular bulb reached above the reference point were determined to be high jugular bulb (HJB) and classified as follows (Fig. 1 C).

Class 1: The apex of the HJB is above the inferior edge of the tympanic ring and below the cochlear basal turn (Fig. 1 C-①).

**Fig. 2:**
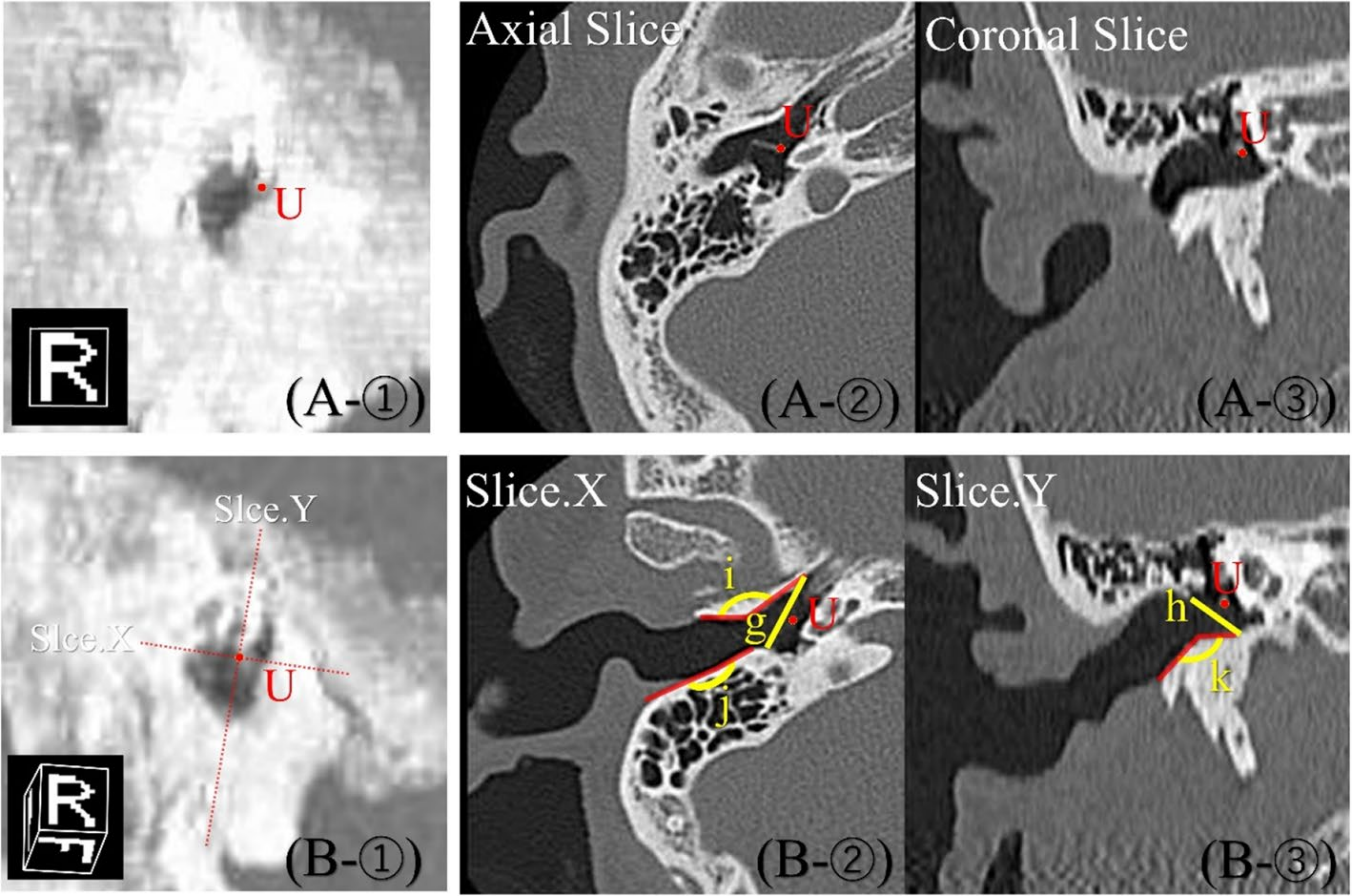
Evaluation of external auditory canal morphology. **A** Shows a 3D (A-①), axial (A-②), and coronal slice (A-③) images of the external auditory canal indicating the tympanic umbo as the point U. **B** Shows a 3D image (B-①), slices passing the short diameter of the bony tympanic ring (slice X, A-②), and the long diameter of the bony tympanic ring (slice Y, A-③) from a view where the tympanic membrane can be confirmed best using MIP processing by rotating visual axis arbitrarily around the point U. B Also shows the antero-posterior (g) and superior-inferior (h) length of the external auditory canal in the bony tympanic ring and the angle of the external auditory canal curvature (anterior wall, i; posterior wall, j; and inferior wall, k). For the angle of the external auditory canal curvature, the angle of the line connecting the point at the entrance of the external auditory canal bone, the most protruding point, and the tympanic ring was measured

Class 2: The apex of the HJB is above the cochlear basal turn and below the lateral semicircular canal (Fig. 1 C-②).

**Fig. 3:**
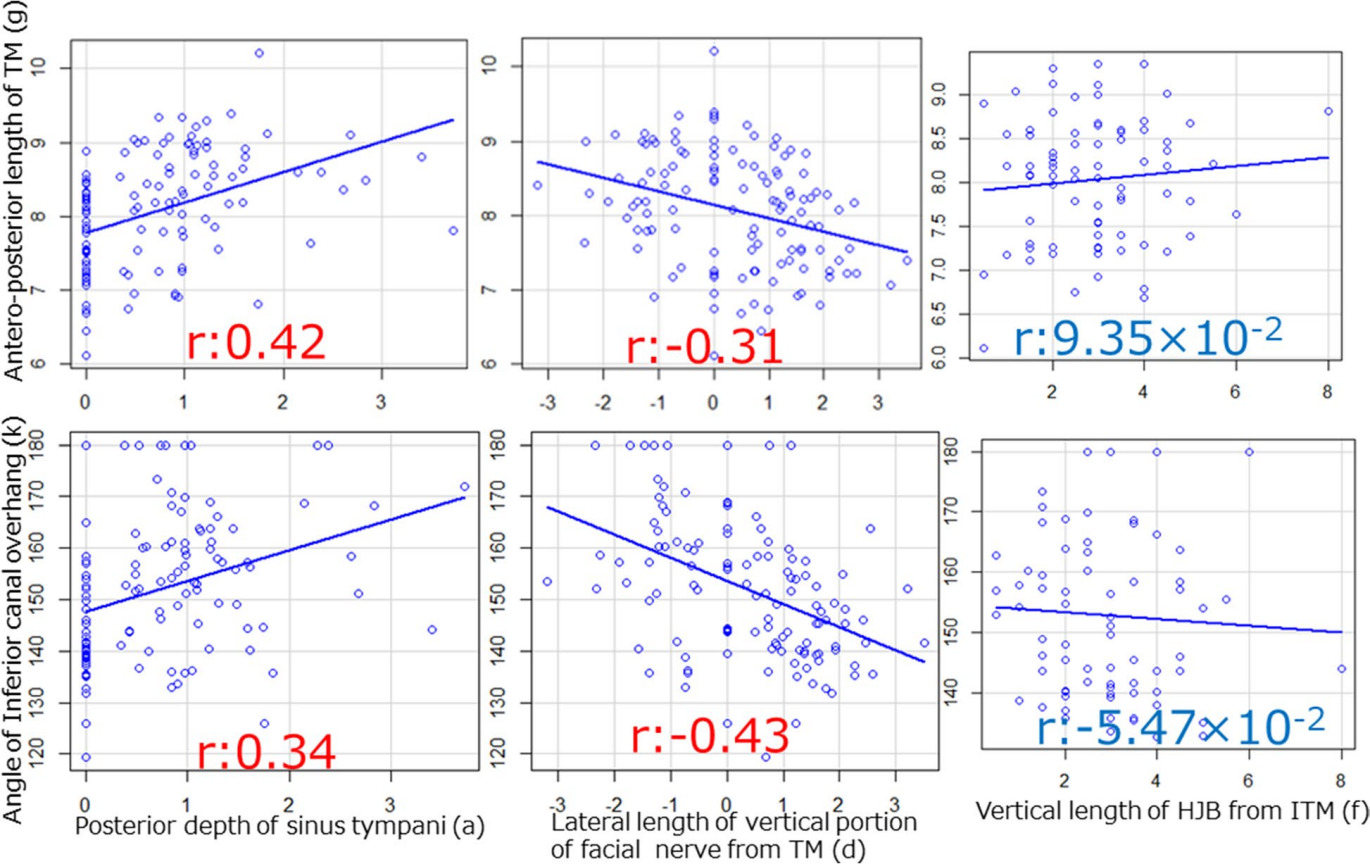
Scatter plot. The antero-posterior length of the bony tympanic ring and the external auditory canal morphology (i.e., the degree of overhang of the inferior wall of the external auditory canal) slightly correlates with the depth of the tympanic sinus, and the lateral process of the vertical portion of the facial nerve

Class 3: The apex of the HJB is above the lateral semicircular canal (Fig. 1 C-③).

Cases without HJB were judged as Class 0. In cases with HJB, the shortest distance (e) from the tympanic membrane to the jugular bulb at the height of the inferior edge of the bony tympanic ring was measured. The height (f) of the jugular bulb was calculated by multiplying the number of slices from the inferior edge of the tympanic ring to the apex of the jugular bulb by the slice width.

### Evaluation of the morphology of the EAC

To create a three-dimensional (3D) image near the tympanic membrane (Fig. 2 A-①), maximum intensity projection (MIP) processing was performed with a slab width of 3 mm centering on the point U indicating the tympanic umbo confirmed from axial (Fig. 2 A-②) and coronal slices (Fig. 2 A-③) of the temporal bone CT images. This image was rotated arbitrarily to set the visual axis direction (Fig. 2B-①) where the tympanic membrane could be confirmed best. Next, on this screen, slices of the short axis of tympanic membrane (Fig. 2B-②) and the long axis of tympanic membrane (Fig. 2B-③) were created. Based on these slices, the antero-posterior (g) and superior-inferior (h) length of the EAC in the bony tympanic membrane were measured. Furthermore, for obtaining the angle of the overhang of the EAC curvature (anterior wall, i; posterior wall, j; and inferior wall, k), the angle of the line connecting the point at the entrance of the EAC, the point of the highest overhang, and the bony tympanic ring were measured. To measure the degree of overhang, the maximum was set to 180 degrees, and it was judged that the smaller the angle, the more severe the overhang.

### Statistical analysis

For statistical analysis, R version 3.6.1 (R Foundation for Statistical Computing, Vienna, Austria) was used, and a *p-*value of < 0.05 was considered statistically significant. The continuous variable was shown by mean ± standard deviation (SD), and the mean value of each class was used as the continuous variable for the classification of the tympanic sinus and jugular bulb. The chronic otitis media (COM) group and the normal group of various measured values ​​were compared using the Mann-Whitney U test. Next, the correlation coefficient between the measured value indicating the morphology of the EAC, which is a continuous variable, and the measured value of various structures in the middle ear was calculated using Spearman’s rank correlation coefficient, and the relationship with each structure was evaluated.

## Results

Table 1 shows the patient characteristics and the results of anatomical measurements. Table 2 shows the results of the comparison between the COM and normal groups for various measurements. Compared to normal ears, ears with COM had a significantly shorter antero-posterior length of the bony tympanic ring and a severe overhang of the inferior wall of the EAC. No significant difference was found in the other measurements of the EAC morphology. Moreover, ears with COM had a shallower tympanic sinus than normal ears, and the vertical portion of the facial nerve tended to run outward. No significant difference was found in the parameters indicating the structure of HJB. Subsequently, the correlation coefficient of the measured values indicating the morphology of the EAC and the structure of the middle ear was calculated (Table 3). A weak correlation was found between the morphology of the EAC, such as the angle of overhang of the inferior wall of the EAC and the antero-posterior length of the bony tympanic ring, and the depth of the tympanic sinus and the lateral process of the vertical portion of the facial nerve. No correlation was found with respect to the height of the HJB (Fig. 3).

**Table 1:** Patient characteristics and anatomical measurements

	MEAN ± SD	N (%)				
Years	65.18 ± 18.77					
Sex (Male)		51(42.5)				
**[Tympanic sinus]**						
Average Class	1.88 ± 0.79	Class1:46(38.3)	Class2:43(35.8)	Class3:31(25.8)		
Posterior depth(a)	0.73 ± 0.78					
Lateral depth(b)	0.27 ± 0.61					
**[Vertical portion of facial nerve]**						
Posterior length from EAC(c)	3.24 ± 0.81					
Lateral length from TM(d)	0.37 ± 1.32					
**[High jugular bulb]**						
Average Class	1.18 ± 1.03	Class0:42(35)	Class1:26(21.7)	Class2:40(33.3)	Class3:12(10)	
Horizontal length from ITM(e)	4.39 ± 2.44					
Vertical length from ITM(f)	2.9 ± 1.36					
**[EAC]**						
Antero-posterior length of TM(g)	8.08 ± 0.77					
Superio-inferior length of TM(h)	7.68 ± 1.07					
Angle of ACOH(i)	165.77 ± 15.6					
Angle of PCOH(j)	164.9 ± 13.59					
Angle of ICOH(k)	151.97 ± 13.79					

*ACOH *Anterior canal overhang, *EAC *External Auditory Canal, *ICOH *Inferior canal overhang, *ITM *Inferior edge of tympanic membrane, *PCOH *Posterior canal overhang, *TM *Tympanic membrane

**Table 2:** Results of statistical analysis of patients with and without chronic otitis media

	COM	Normal	
	MEAN ± SD	MEAN ± SD	*p-*Value
**[Sinus tympani]**			
Average Class	1.64 ± 0.78	1.97 ± 0.78	3.82 × 10^− 2^
Posterior depth(a)	0.39 ± 0.52	0.86 ± 0.82	2.79 × 10^− 3^
Lateral depth(b)	0.28 ± 0.66	0.27 ± 0.6	0.39
**[Vertical portion of facial nerve]**			
Posterior length from EAC(c)	3.08 ± 1.01	3.3 ± 0.72	0.13
Lateral length from TM(d)	1.16 ± 1.11	0.07 ± 0.27	1.02 × 10^− 4^
**[High jugular bulb]**			
Average Class	0.97 ± 0.85	1.26 ± 1.08	0.2
Horizontal length from ITM(e)	4.35 ± 2.51	4.41 ± 2.44	0.79
Vertical length from ITM(f)	2.48 ± 1.27	3.06 ± 1.37	0.15
**[EAC]**			
Antero-posterior length of TM(g)	7.63 ± 0.78	8.25 ± 0.71	9.17 × 10^− 5^
Superio-inferior length of TM(h)	7.27 ± 1.08	7.34 ± 1.03	9.38 × 10^− 3^
Angle of ACOH(i)	166.6 ± 15.47	165.45 ± 15.73	0.72
Angle of PCOH(j)	163.77 ± 13.11	165.32 ± 13.82	0.61
Angle of ICOH(k)	147.1 ± 13.62	153.82 ± 13.47	1.62 × 10^− 2^

Compared to normal ears, COM ears had a significantly shorter bony tympanic ring in terms of antero-posterior length and a severe overhang of the inferior wall of the external auditory canal. There was no significant difference in the measured values ​​indicating other external auditory canal morphology. In ears with COM, tympanic cavity was predominantly shallower than the normal ear, and the vertical portion of the facial nerve tended to run outward. No significant difference was found in the parameters indicating the structure of high jugular bulb

*ACOH *Anterior canal overhang, *COM *Chronic otitis media, *EAC *External auditory canal, *ICOH *Inferior canal overhang, *ITM *Inferior edge of tympanic membrane, * PCOH *Posterior canal overhang, *TM *Tympanic membrane

**Table 3:** Correlation coefficient between external auditory canal morphology and middle ear structure

	Antero-posterior length of TM(g)	Superio-inferior length of TM(h)	Angle of ACOH(i)	Angle of PCOH(j)	Angle of ICOH(k)
**[Sinus tympani]**					
Average Class	0.43	0.23	9.74 × 10^− 3^	5.76 × 10^− 2^	0.35
Posterior depth(a)	0.42	0.12	8.11 × 10^− 2^	-2.41 × 10^− 2^	0.34
Lateral depth(b)	0.24	0.22	5.03 × 10^− 2^	9.41 × 10^− 2^	0.13
**[Vertical portion of facial nerve]**					
Posterior length from EAC(c)	7.01×0.0^− 2^	1.85 × 10^− 3^	0.18	9.26 × 1.0^− 2^	-5.79 × 10^− 2^
Lateral length from TM(d)	-0.31	-0.15	8.67 × 10^− 2^	1.95 × 10^− 2^	-0.43
**[High jugular bulb]**					
Average Class	-3.09 × 10^− 2^	-0.19	2.41 × 10^− 3^	3.87 × 10^− 2^	3.87 × 10^− 2^
Horizontal length from ITM(e)	1.61 × 10^− 2^	8.8 × 10^− 2^	0.1	0.12	6.63 × 10^− 2^
Vertical length from ITM(f)	9.35 × 10^− 2^	-0.29	0.13	-8.49 × 10^− 3^	-5.47 × 10^− 2^

The antero-posterior length of the bony tympanic ring and the morphology of the external auditory canal (i.e., the degree of overhand of the inferior wall of the external auditory canal) were slightly correlated with the depth of the tympanic cavity, and the lateral process of the vertical portion of the facial nerve

*ACOH *Anterior canal overhang, *EAC *External Auditory Canal, *ICOH *Inferior canal overhang, *ITM *Inferior edge of tympanic membrane, *PCOH *Posterior canal overhang, *TM *Tympanic membrane

## Discussion

The EAC is a pathway for transmitting sound to the tympanic membrane [2], but its morphology varies. The curved overhang of the EAC can obstruct the field of vision, affect diagnosis [1], treatment and render operation on the middle ear [3] difficult. Especially when performing middle ear surgery, sometimes it may be necessary to shave the EAC bone to perform the operation. Recent endoscopes have a wide field of view, which could reduce the need for EAC excision [4, 5], but they offer limited operability. When performing procedures or surgical operations via the EAC, it is crucial to understand the condition of vital structures in the middle ear to anticipate the difficulty of surgical operations and prevent secondary damage.

In this study, 3D reconstruction processes MPR (multiplanar reconstruction) and MIP, were used to evaluate the morphology of the EAC. MPR is a process for visualizing an arbitrary cross-sectional image, commonly used in temporal bone CT [6], whereas MIP is a method of projecting the relative maximum value in the region by freely setting the visual axis from the reader’s line of sight to any point in the target structure and determining the arbitrary thickness [7]. These two processes allow the creation of a 3D image since it is possible to visualize multiple slices at the same time. In contrast, the temporal bone CT has limited usefulness because visualization is limited by bone tissue [8]. Regarding the usefulness of MIP in the temporal bone region, evaluation of the auditory ossicles by CT [6, 8] and evaluation of cochlear basal turn by magnetic resonance imaging [9] have been reported; however, only a relatively small number of cases have been reported. In this study, by setting the tympanic umbo as the target point with MIP, moving the visual axis, and confirming the position where the tympanic membrane can be confirmed best, the visual axis that can respond to the curved EAC was determined. By creating MPR slices that pass through the short and long axes of the bony tympanic ring based on this image, it was possible to create slices showing the short and long axes of the bony tympanic ring that also correspond to the curvature of the EAC. Kavita et al. [3] used axial and coronal slices to evaluate EAC curvature in pediatric cases. However, in order to evaluate the morphology of the adult EAC that was curved in a complicated direction due to growth, it was difficult to use axial and coronal slices as horizontal and vertical slices along the long axis of the EAC. MIP is a reconstruction process that can be performed in a relatively short time [6, 8]. Since the visual axis of the reader’s line of sight for the target structure can be freely set in MIP, it is useful for evaluation of the complicatedly curved EAC morphology.

Furthermore, in this study, we investigated the individual anatomical differences of the tympanic sinus, the vertical portion of the facial nerve, and the jugular bulb as important structures during middle ear surgery. The tympanic sinus is the space behind the tympanum [10], a difficult-to-visualize area considered to be a clinically important structure because lesions tend to remain in this space [11].

The facial nerve runs in a complicated manner in the temporal bone, and the closest part to the tympanic ring is the vertical portion [12]. There is no good landmark to estimate the position of the vertical portion of the facial nerve when performing an EAC procedure, and due circumspection is required when performing an EAC excision [13]. The jugular bulb is sometimes present as a high jugular bulb (HJB) near the EAC. It has received significant attention as it may cause massive bleeding during procedures such as tympanostomy [14]. The EAC and these vital structures in the middle ear have large individual anatomical variations, and many studies have been conducted so far. However, it has been reported that it is associated with changes in mastoid cell development mainly due to chronic inflammation. In this study, a slight correlation could be found between the morphology of the bony tympanic ring with a short antero-posterior length and the severe overhang of the inferior wall of the EAC, the shallow tympanic sinus, and the lateral process of the vertical portion of the facial nerve.

For EAC curvature, visual field limitation due to overhangs of the anterior, posterior, and inferior walls of the EAC is clinically important [3]. When the anterior canal overhang (ACOH) is high, the operability of middle ear surgery is often difficult, and ACOH has been discussed for some time; however, there are only a few reports on inferior canal overhang (ICOH) and posterior canal overhang (PCOH). Dedhia et al. [3] first reported that overhang was the most severe in ICOH, and cases with large ICOH overhang were predominantly cases with a history of tympanic tube placement. This finding suggests that the higher the ICOH angle, the more likely it is for eustachian tube dysfunction and otitis media to occur. Furthermore, Park et al. [15] reported that the antero-posterior length of the tympanic membrane was significantly shorter in the COM group than in the normal temporal bone group. However, there is no difference in the superior-inferior length; the antero-posterior direction is more important for the growth of the tympanic membrane after birth than the superior-inferior direction. As such, this suggests that the cavity of the bony EAC may become narrow when its growth is inhibited due to inflammation.

Recently, there has been an increase in reports stating that the growth of mastoid cells is suppressed by chronic inflammation of the middle ear [16–18]. However, regarding changes in vital structures of the middle ear, the tympanic sinus becomes shallow due to chronic inflammation [11, 19] and the vertical portion of the facial nerve runs more outward [20]. Since the tympanic sinus progresses toward the less resistant region during development of mastoid cells, it is thought that suppression of growth of mastoid cells due to chronic inflammation changes the depth of the tympanic sinus [11]. Moreover, the facial nerve has a larger anatomical individual variation in the peripheral vertical portion than in the proximal portion such as the labyrinthine and tympanic segments [13]. Compared to adults, it runs more outward in children, and its position changes as one grows [21]. In cases where growth of mastoid cells is suppressed, the vertical portion of the facial nerve runs more anteriorly and laterally. The vertical portion of the facial nerve also suppresses the medio-posterior movement with growth by suppressing the mastoid growth [20].

From the results obtained in this study, anatomical morphologies such as the EAC, tympanic sinus, and the vertical portion of the facial nerve that change after birth, may change in conjunction to some extent. Similar to suppression of the growth of mastoid cells by chronic inflammation, morphological changes may occur mainly due to chronic inflammation. Therefore, if there are findings such as a short antero-posterior length of the bony tympanic ring or a severe overhang of the inferior wall of the EAC during treatment or surgery via the EAC, it can be predicted that the tympanic sinus is shallow, and the vertical part of the facial nerve is likely to run outward. Temporal CT is often performed prior to the middle ear surgery; however, the fact that these structures can be predicted from the EAC morphology is meaningful in assuming the difficulty of preventing secondary damage and removing cholesteatoma in surgery.

From this study, a favorable correlation between the height of HJB and the morphology of the EAC could not be obtained. There have been reports that the height of the jugular bulb is also affected by the growth of mastoid cells, and that better the growth, the higher the apex of the jugular bulb [22, 23]. Therefore, it was speculated that there may be a correlation between the EAC morphology and the height of the jugular bulb. However, in this study, the height of non-HJB cases classified as class 0 could not be attributed to why the correlation with the height of HJB and the class classification could not be obtained, and it is possible that only the height of the case defined as HJB was measured. Although future studies are needed, the relationship between clinically significant HJB, and its height and EAC morphology is low.

There are some limitations in this study. First: since the temporal bone CT used in this anatomical examination used a slice with a width of 0.5 mm, the reliability may be low for measurements of 0.5 mm or less. Second: in this study, as in previous reports, ears with COM tended to have shorter bony tympanic rings, severe overhangs in the lower wall of the EAC, and shallower tympanic sinus, and the vertical portion of facial nerves tended to run laterally compared to normal ears. This study further suggested that there may be a correlation between changes in EAC morphology and changes in middle ear structures. However, it was not possible to elucidate how these changes affect the duration and extent of chronic inflammation in this study. Thus a prospective study that includes the duration of chronic inflammation is needed in the future.

## Conclusion

During treatment via the EAC, when there were findings of short antero-posterior length of the bony tympanic ring and a severe overhang of the inferior wall of the EAC, it is highly possible that the tympanic sinus is shallow, and the vertical portion of the facial nerve runs more outward. By recognizing these in advance, it is possible to predict the difficulty of operation on the tympanic sinus and reduce the risk of facial nerve damage during EAC excision.

## Data Availability

The authors declare availability of data and materials.
